# Effects of clear corneal incision location and morphology on corneal surgically induced astigmatism and higher-order aberrations after ICL V4c implantation

**DOI:** 10.3389/fmed.2024.1491901

**Published:** 2024-11-06

**Authors:** Jun Wang, Xiaoying He, Qin He, Jin Han, Zixuan Yang, Xuze Wang, Wei Han

**Affiliations:** ^1^Department of Eye Center, The Second Affiliated Hospital, Zhejiang University School of Medicine, Hangzhou, China; ^2^Department of Ophthalmology, The First Affiliated Hospital, Zhejiang University School of Medicine, Hangzhou, China

**Keywords:** implantable collamer lens, clear corneal incision, surgically induced astigmatism, higher-order aberrations, anterior segment optical coherence tomography

## Abstract

**Purpose:**

To evaluate the effects of clear corneal incision (CCI) location and morphology on corneal surgically induced astigmatism (SIA) and higher-order aberrations (HOAs) in patients receiving implantable collamer lens (ICL V4c) implantation.

**Methods:**

This retrospective study classified right eyes that underwent ICL implantation into two groups based on temporal or superior CCI. The Pentacam HR analyzer was used to measure the corneal astigmatism and HOAs. Analysis of the clear corneal incision (CCI) morphology, including incision width (Angle-W), incision length (IL), incision angles (Angle-En/Ex), and distance from the incision to corneal apex (Dis-En/Ex), was conducted using anterior segment optical coherence tomography (AS-OCT).

**Results:**

There were 75 eyes in the temporal CCI group and 47 eyes in the superior CCI group. Both groups showed satisfactory safety and efficacy postoperatively. In the temporal CCI group, Dis-En and Dis-Ex were considerably longer, whereas the superior CCI group displayed a significantly wider Angle-W. The anterior and posterior corneal SIA were comparable in both groups. Anterior corneal SIA was significantly correlated to Dis-En and Dis-Ex in the superior CCI group. Superior CCI caused a notable rise in corneal Z (3, 3), while temporal CCI led to increased Z (3, 1). CCI morphology was correlated to corneal Z (4, −4) and Z (4, 4) in the superior CCI group.

**Conclusion:**

CCI locations caused slight variations in postoperative corneal SIA and HOAs following ICL implantation. Optimal postoperative visual outcomes may be better achieved with a CCI design featuring an increased distance from the corneal centroid and a decreased Angle-W.

## Introduction

Implantation of the Visian Implantable Collamer lens (ICL, STAAR Surgical) provides significant benefits across a broad spectrum of daily activities and serves as a life-changing intervention for numerous high myopia patients (1–5). ICL implantation is now considered a feasible refractive surgery option for moderate and low myopia, given its rapid recovery, excellent refractive outcomes, and reversibility, while avoiding corneal ablation (6). Therefore, heightened standards are essential for the visual and refractive outcomes following ICL implantation.

Surgically induced astigmatism (SIA) resulting from a clear corneal incision (CCI) during surgery is inevitable and represents a crucial factor influencing postoperative visual and refractive outcomes in ICL implantation. The refractive power is consistently diminished along the meridian by corneal incisions, resulting in changes to corneal astigmatism (7, 8). When preparing for the placement of an ICL, different methods are used to adapt to the limited size of the ICL and various eye shapes, with the aim of achieving an ideal vault for long-term safety assurance (9). Combining particular CCI locations with distinct ICL placement orientations serves to minimize rotational angles in surgery, ultimately lowering operation time and mitigating intraoperative complications. Temporal and superior CCIs are widely employed as corneal incision locations in clinical practice. A study by Kamiya et al. (10) revealed a marked increase in corneal astigmatism post-ICL surgery among individuals in the temporal CCI group, while a reduction was noted in the vertical CCI group. Further investigation is required to understand the differences in corneal SIA induced by various CCI locations and the influencing factors to achieve optimal visual and refractive outcomes after ICL placement.

Apart from the effects of CCI sites, corneal SIA following surgery is influenced by parameters such as incision width, incision length, and the distance from the incision to the corneal apex. Numerous studies have established that wider and longer CCIs located in proximity to the corneal apex yield greater corneal SIA after cataract surgery (11–13). ICL implantation differs from cataract surgery in that it is generally undertaken on young adults with superior recovery capabilities and differing corneal astigmatism (14, 15). Additionally, ICL implantation omits the need for phacoemulsification procedures and has relatively less effect on the cornea, potentially offering a more precise assessment of the effect of CCI on corneal astigmatism.

Besides corneal SIA, the existence of increased higher-order aberrations (HOAs) post-refractive surgery significantly influences visual acuity and leads to complications such as glare, ghosting, and compromised night vision (16, 17). Although the introduction of HOAs following ICL implantation is notably lower than that of corneal refractive surgery, there are still observed increases in total HOAs, trefoil, and spherical aberrations (SA) (18–20). Several factors, including corneal incisions, the ICL lens, and the eccentricity and tilt of the ICL lens, are likely responsible for the heightened presence of HOAs post-ICL surgery. However, to the best of our knowledge, still, no studies have reported the correlation between corneal morphological features and corneal HOAs/SIA following temporal or superior corneal incisions during ICL implantation.

In this study, patients were categorized into two groups based on the location of CCIs: the temporal CCI group and the superior CCI group. The visual and refractive outcomes, CCI features, corneal SIA, and corneal HOAs were analyzed for the two groups. Furthermore, associations between CCI characteristics and the levels of corneal SIA and HOAs were investigated in both groups. These findings provide valuable insights into optimizing ICL surgical techniques regarding the corneal incision creation, appropriate ICL refraction selection, and ICL implantation procedure design.

## Materials and methods

This retrospective investigation involved the analysis of data from patients who received same-day bilateral phakic intraocular lens implantation at the Eye Center, Second Affiliated Hospital, Zhejiang University, Hangzhou, China between April 2021 and March 2023. Informed consent was obtained from all participants. The study received approval from the hospital’s ethics committee and adhered to the principles outlined in the Declaration of Helsinki.

To prevent any confusion from aberration symmetry and Zernike polynomial reconstruction in the fellow eyes, only the right eyes were considered in this study (21, 22). The inclusion criteria were as follows: (i) age between 18 and 45 years, (ii) myopia ranging from −0.50 to −18.00 D with or without astigmatism (−0.50 to −6.00 D), and myopic spherical equivalent refraction (SER) > −18.00 D, (iii) stable refraction for at least 2 years with no increase greater than 0.50 D per year, (iv) anterior chamber depth (ACD) ≥ 2.80 mm, (v) corneal endothelial cell density (ECD) ≥ 2000 cell/mm^2^, (vi) anterior corneal astigmatism<3.00 D. Patients with a history of ocular trauma or surgery, coexisting ocular diseases such as severe dry eyes, corneal scar, keratoconus or topographic suspicions of keratoconus, cataract, glaucoma or suspected glaucoma, uveitis and retinopathy; pregnancy or breastfeeding; systematic diseases that may interfere with corneal healing such as diabetes mellitus and connective tissue disorders were excluded from the study.

Prior to surgery, all patients underwent a comprehensive ophthalmic examination that included slit-lamp biomicroscopy, intraocular pressure (IOP), manifest and cycloplegic refraction, uncorrected distance visual acuity (UDVA), corrected distance visual acuity (CDVA), ECD, tear break-up time, corneal tomography (Pentacam HR, Oculus Optikgerate GmbH), optical biometry (IOLMaster 700, Carl Zeiss Meditec AG), ultrasound B scan, fundus photography, and ultrasound biomicroscopy (UBM, Model SW 3200 L, Tianjin Suowei Electronic Technology Co., Ltd.). Prior to the preoperative evaluation, patients were directed to cease wearing soft contact lenses for at least 1 week; rigid gas-permeable contact lenses for at least 1 month; and orthokeratology lenses for at least 3 months.

Through preoperative assessments and the STAAR surgical calculator, the surgeon determined the size and placement orientation of the ICL. Toric implantable collamer lenses (TICL) would be chosen if subjective astigmatism was over 0.75 D or if correcting astigmatism could enhance CDVA by more than 2 lines.

### Surgical procedures

All surgeries were conducted by the same experienced refractive surgery specialist (W.H.) under topical anesthesia and aseptic conditions. Dilating and cycloplegic agents were administered to the patients on the day of surgery. Following topical anesthesia, a temporal or superior biplane CCI was made using a 3.0-mm gemstone scalpel, along with a 0.6-mm side port incision positioned at 90° from the main port as per ICL placement orientation design. The superior incision was implemented based on the ICL implantation orientation being ≤22° from the vertical meridian, while the temporal incision was carried out at an angle of ≤22° from the horizontal meridian. Subsequently, an injector cartridge was used to implant a model V4c ICL through the main incision, while injecting a moderate viscoelastic device into the anterior chamber. An ICL manipulator was employed to position each flexible footplate in the ciliary sulcus, followed by rotation of the ICL to achieve its intended orientation. Finally, a balanced salt solution was used for washing out the viscoelastic device from the anterior chamber without suturing any of the incisions. A final verification of lens centration and alignment was performed after hydrating all incisions.

After the surgical procedure, all patients were administered tobramycin dexamethasone eye drops four times daily for 1 week, followed by 0.1% fluorometholone eye drops four times daily for 3 weeks. Additionally, artificial tears were applied four times daily for a duration of 3 months. Follow-up examinations were scheduled at intervals of 1 week, 1 month, 3 months, 6 months, 1 year, and annually post-surgery. Each clinical visit included slit-lamp biomicroscopy, IOP, UDVA, and CDVA, as well as an assessment of vault and CCI features using anterior segment optical coherence tomography (AS-OCT, SS-1000 CASIA, Tomey Corp.). Corneal tomography was undertaken after a minimum three-month postoperative period.

### Pentacam assessments

The Pentacam measurement was performed by experienced technicians as described in our previous work (23). The following data were extracted from Pentacam measurement: (i) keratometry values (by magnitude and meridian) for both anterior and posterior corneal surfaces, (ii) ACD, (iii) central corneal thickness (CCT), and (iv) 3rd to 4th order Zernike coefficients, as well as root-mean-square (RMS) of total HOAs (tHOAs) over central 4- and 6-mm diameter zones on total cornea, anterior and posterior corneal surfaces. Coma was calculated as the RMS of Z (3, −1) and Z (3, 1). Trefoil was calculated as the RMS of Z (3, −3) and Z (3, 3). The 2nd astigmatism was calculated as the RMS of Z (4, −2) and Z (4, 2). Tetrafoil was calculated as the RMS of Z (4, −4) and Z (4, 4).

### AS-OCT assessments

**Figure 1:**
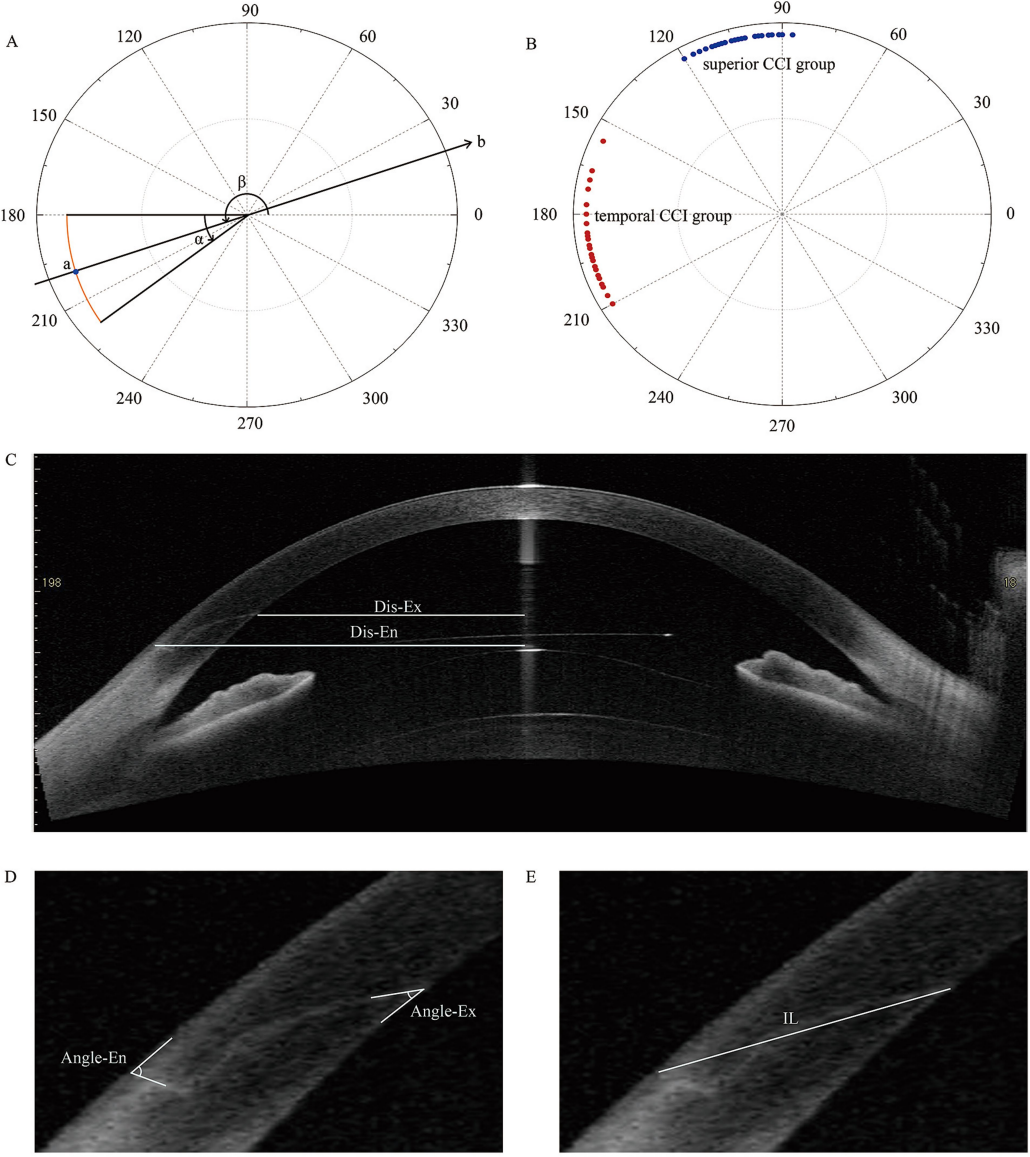
**(A)** Pattern of clear corneal incisions (CCIs). The orange line represents a temporal CCI; point a: the central point of the CCI; *α*: the angle corresponding to the width of the CCI (Angle-W); *β*: the angle of the central point of CCI; line b: the meridian of cross-sectional images of CCI. **(B)** Distribution of CCI sites (central point) in temporal (red) and superior (blue) groups. **(C–E)** Assessments of CCI features on AS-OCT images. Dis-En, the distance from incision entry to central cornea; Dis-Ex, the distance from incision exit to central cornea; Angle-En, angle between incision and corneal epithelium; Angle-Ex, angel between incision and corneal endothelium; IL, incision length.

The swept source AS-OCT assessments were conducted by experienced technicians, following the methodology described in our previous publication (12). AS-OCT images capturing the cross-sections of CCI were acquired along the meridian originating from the incision center and spanning the corneal vertex, then subjected to analysis (Figure 1). The package caliper tool provided by Tomey Measurement software (Tomey Corp.) and Image-J software was utilized to measure the following incision parameters: (i) angle corresponding to the corneal incision width (Angle-W), (ii) straight distance from incision entry to exit (incision length, IL), (iii) angle between the incision and corneal epithelium/endothelium (Angle-En and Angle-Ex), (iv) distances from the central cornea to the incision entry (Dis-En) and exit points (Dis-Ex). To secure the accuracy and consistency of measurements, two independent observers were responsible for conducting all manual measurements. Each observer executed three measurements, and the mean value was selected as the representative value for each observer. The final result was established by averaging the values reported by the two observers.

### Statistical analysis

SIA vectors were calculated with the method described by Alpins & Goggin (24–26). The superior incision group involved eyes with 60° < *β* ≤ 120°and the temporal incision group involved eyes with 150° < *β* ≤ 210° (Figure 1). Statistical analysis was performed using SPSS (version 21.0, IBM Corp.). Data was presented as mean ± standard deviation (SD). The Shapiro–Wilk test was used to assess data normality. Pearson’s chi-square test was used for categorical variables, and the Student’s *t*-test or Mann–Whitney *U* test was used for group-wise comparisons. A paired-sample *t*-test was used to compare pre- and post-operative parameters. Correlation analysis between corneal HOAs, SIA, and CCI features employed either Pearson’s or Spearman’s correlation tests. To address multiple testing issues in group-wise comparisons, intra-group comparisons, and correlation analyses, we applied false discovery rate (FDR) correction at a level of less than 0.05 (27).

## Results

**Figure 2:**
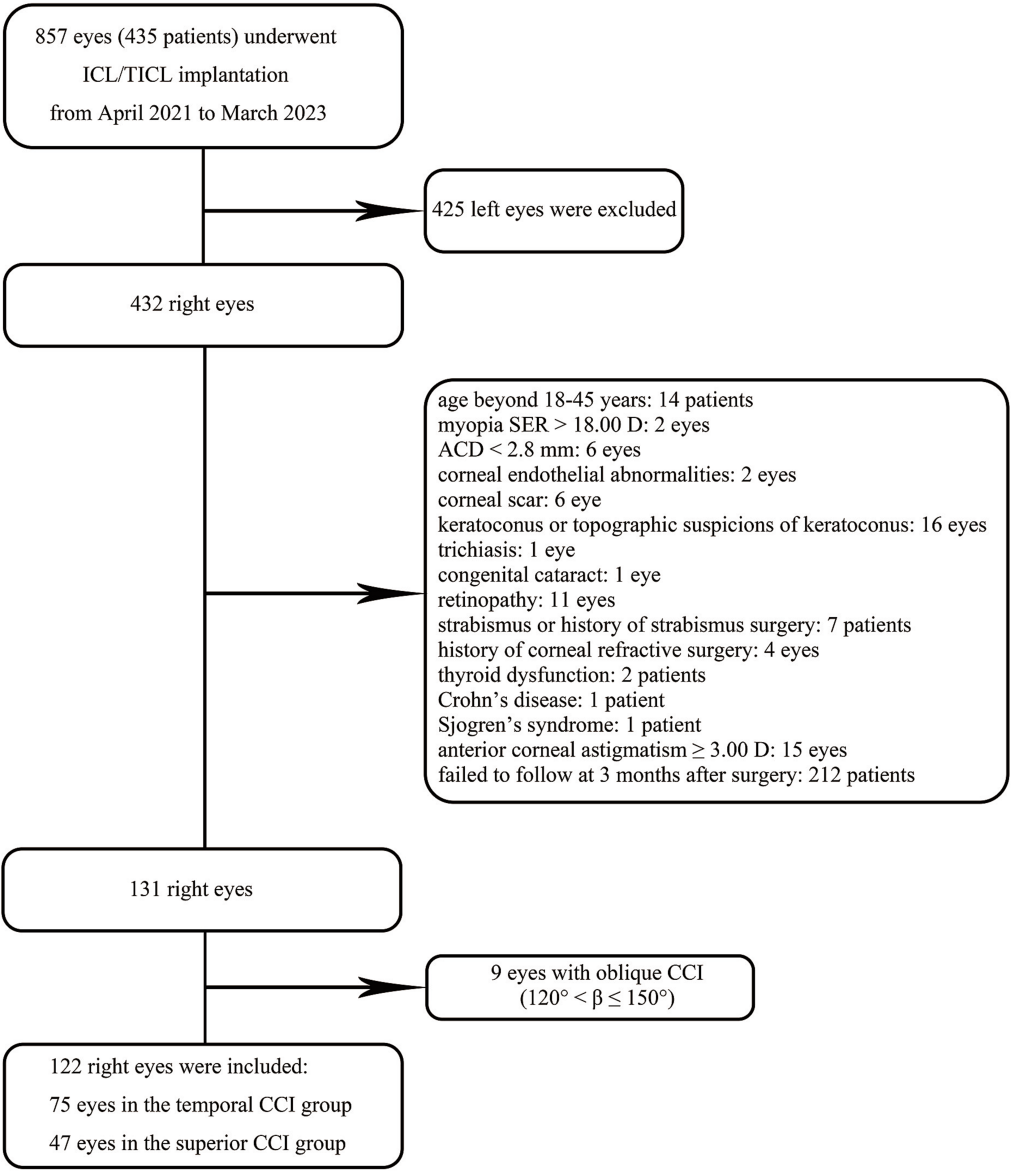
The flow chart depicting the inclusion and exclusion criteria for research subjects.

Following the application of the specified criteria, 75 eyes met the requirements for inclusion in the temporal CCI category, with 47 eyes meeting the criteria for the superior CCI category (Figure 2). The distribution of corneal CCI locations (central point) in both groups is presented in Figure 1B.

The demographic and baseline data of the temporal and superior groups are presented in Table 1. A significantly higher proportion of TICL was observed among individuals in the temporal CCI group (*p* < 0.001). Both groups had comparable baseline clinical characteristics, except for cylindrical diopter (*p* < 0.001) and ACD (*p* = 0.003). Furthermore, the postoperative vault in the superior CCI group was significantly lower than that observed in the temporal CCI group (*p* = 0.009) (Table 1).

**Table 1:** Preoperative and postoperative basic parameters.

Parameter	Temporal (*n* = 75)	Superior (*n* = 47)	*P*-value
Age (y)	27.05 ± 6.08	28.21 ± 6.19	0.292
Sex, *n* (%)			
Male	26 (34.67)	10 (21.28)	0.115
Female	49 (65.33)	37 (78.72)	
ICL type, *n* (%)			
ICL	16 (21.33)	31 (65.96)	<0.001^*^
TICL	59 (78.67)	16 (34.04)	
ICL size, *n*			
12.1/12.6/13.2/13.7	14/29/29/3	5/21/18/3	NA
Preoperative			
Spherical diopter (D)	−9.31 ± 2.43	−8.85 ± 2.22	0.346
Cylindrical diopter (D)	−1.34 ± 0.76	−0.82 ± 0.84	<0.001^†^
Spherical equivalence (D)	−9.98 ± 2.48	−9.26 ± 2.3	0.110
IOP (mmHg)	14.79 ± 2.76	13.92 ± 2.79	0.094
ACD (mm)	3.30 ± 0.24	3.17 ± 0.24	0.003^†^
WTW (mm)	11.68 ± 0.40	11.52 ± 0.41	0.038
CCT (μm)	518.24 ± 33.18	511.81 ± 34.86	0.309
ECD (cell/mm^2^)	2736.47 ± 201.41	2687.68 ± 245.85	0.236
Postoperative			
IOP (mmHg)	15.04 ± 2.61	14.12 ± 2.70	0.115
Vault (μm)	633.41 ± 248.46	499.24 ± 221.7	0.009^※^

ACD, anterior chamber depth; AL, axial length; CCT, central corneal thickness; ECD, endothelial cell density; IOP, intraocular pressure. ^*^Significant (*p* < 0.0167) after correction for multiple comparisons (*n* = 3) based on false discovery rate (FDR) for preoperative basic parameters; ^†^significant (*p* < 0.0188) after correction for multiple comparisons (*n* = 8) based on FDR for postoperative basic parameters; ^※^Significant (*p* < 0.0250) after correction for multiple comparisons (*n* = 2) based on FDR for postoperative basic parameters.

### Visual acuity and refractive outcomes

The UDVA (LogMAR) was −0.003 ± 0.071 and 0.002 ± 0.063 in the temporal CCI group and superior CCI group, respectively (*p* = 0.278). The CDVA (logMAR) improved to −0.012 ± 0.043 (*p* = 0.0002) in the temporal group and − 0.015 ± 0.047 (*p* = 0.005) in the superior CCI groups, respectively, with no significant difference between groups observed either preoperatively (*p* = 0.675) or postoperatively (*p* = 0.675). The efficacy index (a ratio of postop-UDVA/preop-CDVA) was 1.059 ± 0.200 in the temporal CCI group and 1.029 ± 0.154 in the superior CCI group (*p* = 0.170). Similarly, the safety index, a ratio of postop-CDVA/preop-CDVA, were also comparable between the two groups with values of 1.073 ± 0.173 for the temporal CCI group and 1.070 ± 0.153 for the superior CCI group (*p* = 0.920). The scatterplot of attempted and achieved SER, visual acuity, and postoperative SER in both groups is shown in Supplementary Figure S1.

### Corneal keratometry data

With an FDR level of 0.05, the threshold for significant difference between pre- and postoperative measurements was set at 0.0417 in the temporal CCI group and 0.0250 in the superior CCI group (Supplementary Table S1). In the temporal CCI group, K_f_ exhibited a significant decrease on both anterior and posterior corneal surfaces (*p* < 0.00001), while K_s_ remained stable after surgery (*p* > 0.0417). Moreover, there was a significant increase in corneal astigmatism magnitude on both anterior and posterior corneal surfaces following surgery (*p* < 0.0417). Conversely, only K_s_ and corneal astigmatism magnitude of the posterior corneal surface showed a significant decrease in the superior CCI group (*p* < 0.0250).

With an FDR level of 0.05, the threshold for significant difference between the temporal and superior CCI groups was set at 0.0083 for preoperative measurements and 0.0125 for postoperative measurements (Supplementary Table S1). No statistically significant difference was observed between the groups preoperatively (*p* > 0.0083), while the corneal astigmatism magnitude of both anterior and posterior corneal surfaces showed a significantly smaller value in the superior CCI group after surgery (*p* < 0.0125).

### CCI features assessments

After correction for multiple comparisons (*n* = 6) based on FDR, a *p-*value less than 0.0250 was considered as statistically significant (Table 2). The Angle-W in the superior CCI group exhibited a significantly larger value compared to that in the temporal CCI group (*p* < 0.001). Conversely, larger values of Dis-Ex and Dis-En were observed in the temporal CCI group (*p* < 0.00001). No significant difference was found between groups regarding IL, Angle-Ex, and Angle-En (*p* > 0.0250).

**Table 2:** Clear corneal incision features and surgically induced astigmatism.

	Temporal	Superior	*P-*value
CCI features			
Angle-W (°)	36.05 ± 3.3	38.4 ± 4.72	<0.001^*^
Dis-Ex (mm)	4.84 ± 0.36	4.44 ± 0.38	<0.00001^*^
Dis-En (mm)	5.99 ± 0.28	5.64 ± 0.37	<0.00001^*^
IL (mm)	1.66 ± 0.24	1.67 ± 0.24	0.403
Angle-Ex (°)	30.04 ± 5.58	29.69 ± 8.36	0.800
Angle-En (°)	76.72 ± 13.03	78.53 ± 10.38	0.375
Corneal SIA			
Anterior corneal surface			
SIA magnitude (D)	0.40 ± 0.23	0.38 ± 0.18	0.866
Centroid SIA (magnitude @ axis)	0.23@116.58	0.27@27.92	NA
Flattening effect (D)	−0.12 ± 0.28	0.19 ± 0.26	< 0.00001^†^
Torque (D)	−0.13 ± 0.32	0.09 ± 0.25	< 0.001^†^
Absolute torque (D)	0.27 ± 0.21	0.22 ± 0.15	0.321
Posterior corneal surface			
SIA magnitude (D)	0.11 ± 0.05	0.10 ± 0.06	0.724
Centroid SIA (magnitude @ axis)	0.06@119.30	0.04@21.64	NA
Flattening effect (D)	−0.04 ± 0.08	0.06 ± 0.07	< 0.00001^†^
Torque (D)	−0.03 ± 0.08	0.01 ± 0.08	< 0.001^†^
Absolute torque (D)	0.06 ± 0.05	0.06 ± 0.05	0.396

Angle-W, angle corresponding to the corneal incision (incision width); CCI, clear corneal incision; D, diopter; Dis-En, the distance from incision entry to central cornea; Dis-Ex, the distance from incision exit to central cornea; IL, the straight distance from incision entry to exit (incision length); SIA, surgically induced astigmatism. ^*^Significant (*p* < 0.0250) after correction for multiple comparisons (*n* = 6) based on false discovery rate (FDR); ^†^significant (*p* < 0.0250) after correction for multiple comparisons (*n* = 8) based on FDR.

### Corneal SIA assessments

The anterior and posterior corneal SIA in the temporal and superior CCI groups are presented in Supplementary Figure S2. After adjusting for multiple comparisons, there were no significant differences observed in the magnitude of corneal SIA or absolute torque on both the anterior and posterior corneal surfaces between the two groups (*p* > 0.0250) (Table 2). However, a greater flattening effect was noted on both the anterior and posterior corneal surfaces in the superior CCI group, while a larger torque was observed on both surfaces in the temporal CCI group (*p* < 0.0250).

### Corneal HOAs analysis

For group-wise comparisons, there were no significant differences observed between the temporal and superior CCI groups preoperatively over 4- and 6-mm optical zones (*p* > 0.0012) (Supplementary Tables S2, S3). However, significant differences were found between the groups in total corneal Z (4, 4), anterior and posterior corneal Z (3, 3), posterior corneal tHOAs, trefoil, and tetrafoil over 4-mm optical zone after surgery (*p* < 0.0071) (Supplementary Table S4). For the 6-mm optical zone, significant differences were observed between groups in total, anterior and posterior corneal Z (3, 3) and posterior corneal tetrafoil (*p* < 0.0048) (Supplementary Table S5).

#### Coma, trefoil, tetrafoil, 2nd astigmatism, and tHOAs

For intra-group comparisons, with an FDR level of 0.05, the cut-off for significant changes after surgery was *p* < 0.0071 in the temporal CCI group over the 4-mm zone, *p* < 0.0095 in the superior CCI group over the 4-mm zone, *p* < 0.0262 in the temporal CCI group over the 6-mm zone, and *p* < 0.0167 in the superior CCI group over the 6-mm zone.

**Figure 3:**
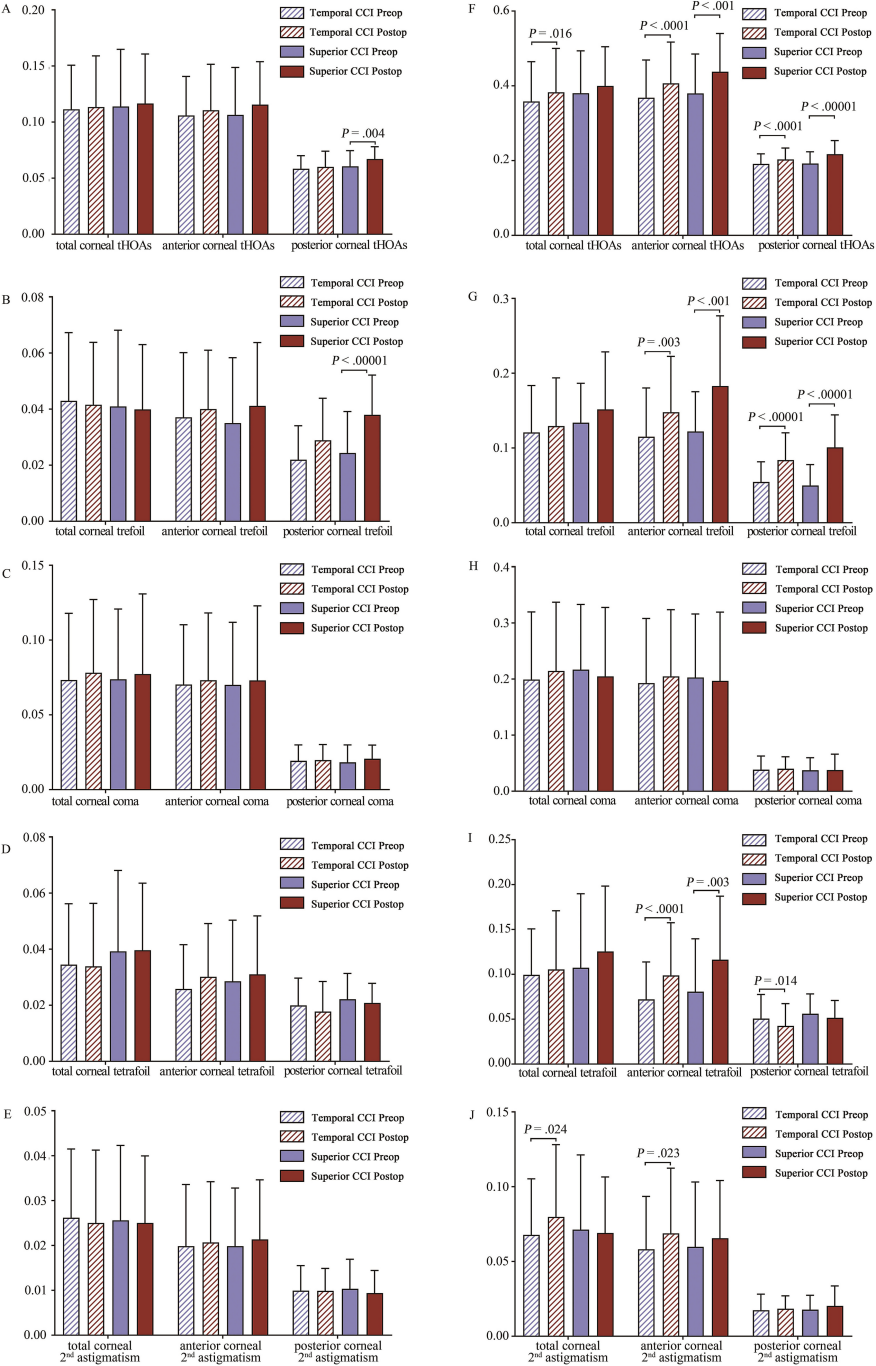
Corneal total and combined higher order aberrations (HOAs) on total, anterior, and posterior corneal surfaces. **(A)** Corneal tHOAs over 4-mm optical zone; **(B)** Corneal trefoil over 4-mm optical zone; **(C)** Corneal coma over 4-mm optical zone; **(D)** Corneal tetrafoil over 4-mm optical zone; **(E)** Corneal 2nd astigmatism over 4-mm optical zone; **(F)** Corneal tHOAs over 6-mm optical zone; **(G)** Corneal trefoil over 6-mm optical zone; **(H)** Corneal coma over 6-mm optical zone; **(I)** Corneal tetrafoil over 6-mm optical zone; **(J)** Corneal 2nd astigmatism over 6-mm optical zone. With a false discovery rate (FDR) level of 0.05, the cut-off for significant changes after surgery was *p* < 0.0071 in the temporal CCI group over the 4-mm zone, *p* < 0.0095 in the superior incision group over the 4-mm zone, *p* < 0.0262 in the temporal CCI group over the 6-mm zone, and *p* < 0.0167 in the superior CCI group over the 6-mm zone. The *p*-value is shown when there is a significant difference between pre- and post-operative data.

**Figure 4:**
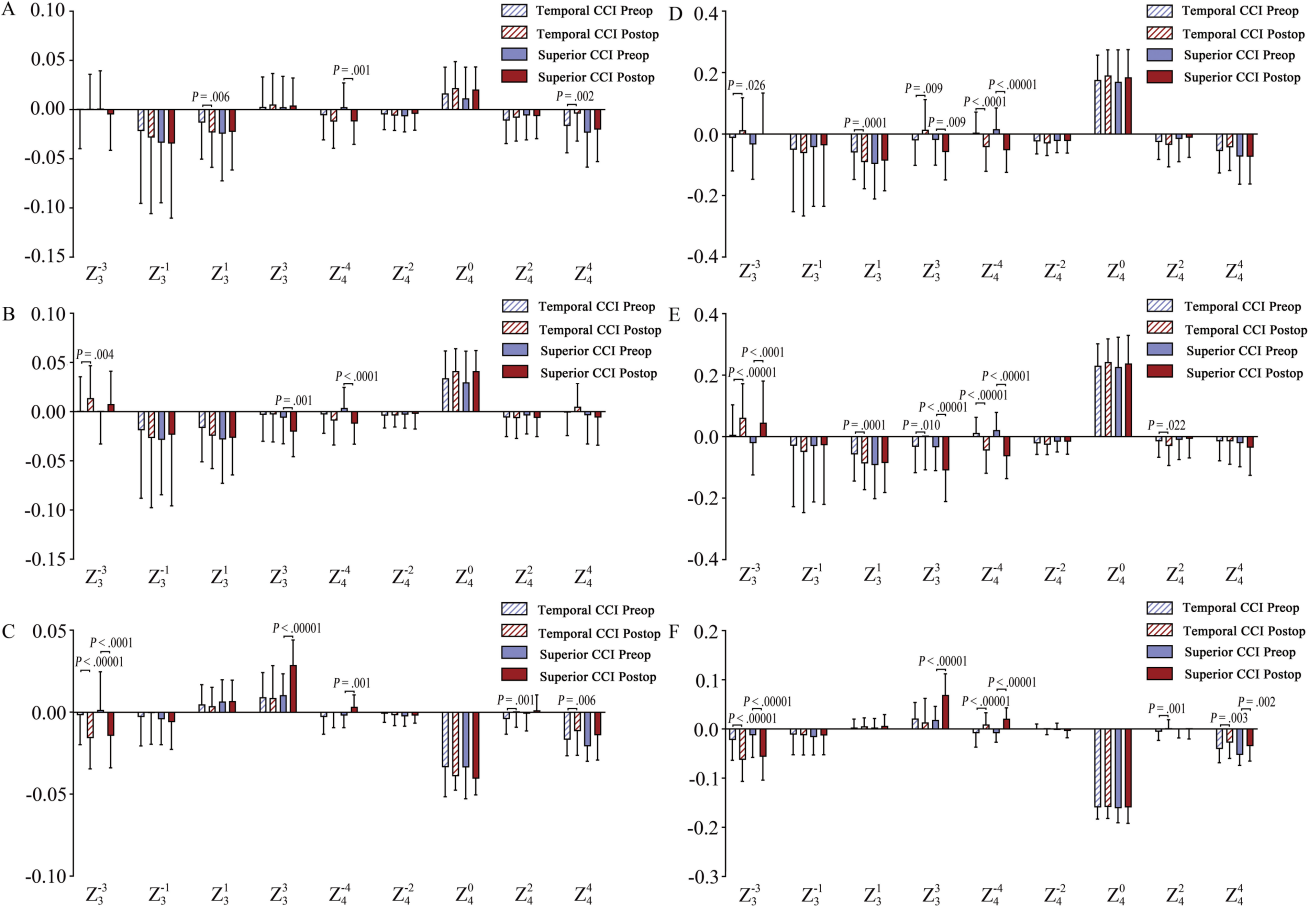
Individual Zernike HOAs analysis. **(A,D)** Individual Zernike terms for the total corneal surface within a 4-mm and 6-mm optical zone, respectively; **(B,E)** Individual Zernike terms for the anterior corneal surface within a 4-mm and 6-mm optical zone, respectively. **(C,F)** Individual Zernike terms for the posterior corneal surface within a 4-mm and 6-mm optical zone, respectively. Statistical Significance: A false discovery rate (FDR) of 0.05 was used to determine the threshold for significant changes post-surgery. The significance thresholds (*p*-values) after surgery were as follows: 4-mm Zone: Temporal CCI group: *p* < 0.0071, Superior CCI group: *p* < 0.0095; 6-mm Zone: Temporal CCI group: *p* < 0.0262; Superior CCI group: *p* < 0.0167. *p*-value display: the *p*-value is indicated for instances where there is a statistically significant difference between pre-operative and post-operative data.

The results depicted in Figure 3 illustrated the variations in a pre- and postoperative coma, trefoil, tetrafoil, 2nd astigmatism, and tHOAs of the total cornea, and anterior and posterior corneal surfaces. For the 4-mm analytical zone, only the superior CCI group showed a significant increase in posterior corneal tHOAs (*p* = 0.004) and trefoil (*p* < 0.00001) after surgery (Figures 4A,B). For the 6-mm analytical zone, both temporal and superior CCI groups exhibited increased anterior and posterior corneal tHOAs, while an increase in total corneal tHOAs (*p* = 0.016) was observed solely in the temporal CCI group (Figure 3F). Both temporal (*p* < 0.0262) and superior (*p* < 0.0167) CCI groups demonstrated a significant increase in anterior and posterior corneal trefoil, whereas corneal coma remained stable in both groups (Figures 3G,H). A significant increase in anterior corneal tetrafoil was found for both temporal (*p* < 0.0001) and superior (*p* = 0.003) CCI groups, whereas a decrease in posterior corneal tetrafoil was observed specifically within the temporal CCI group (*p* = 0.014) (Figure 3I). Furthermore, there was a significant increase noted for 2nd astigmatism within the temporal CCI group for total cornea (*p* = 0.024) and anterior corneal surface (*p* = 0.024) (Figure 3J).

#### Individual Zernike HOAs analysis over the 4-mm analytical zone

For the total cornea, a significant increase in Z (3, 1) (negatively) (*p* = 0.006) and a decrease in Z (4, 4) (*p* = 0.002) were observed exclusively in the temporal CCI group, while significant changes in Z (4, −4) (*p* = 0.001) were only found in superior CCI group (Figure 4A). For the anterior corneal surface, a significant increase in Z (3, −3) (*p* = 0.004) was identified within the temporal CCI group, whereas significant changes in Z (3, 3) (*p* = 0.001) and Z (4, −4) (*p* < 0.0001) were solely present within the superior CCI group (Figure 4B). Regarding the posterior corneal surface, both temporal (*p* < 0.00001) and superior (*p* < 0.0001) CCI groups exhibited a significant increase in Z (3, –3) (negatively); However, only the temporal group showed a notable decrease in Z (4, 2) (*p* = 0.001) and Z (4, 4) (*p* = 0.006), while only the superior group demonstrated a substantial increase in Z (3, 3) (*p* < 0.00001) and Z (4, –4) (positively) (*p* = 0.001) (Figure 4C).

#### Individual Zernike HOAs analysis over the 6-mm analytical zone

For the total cornea, significant changes in Z (3, 3) (positive shift) and Z (4, −4) (negative increase) were observed in both the temporal (*p* < 0.0262) and superior (*p* < 0.0167) CCI groups. However, only the temporal CCI group showed significant changes in Z (3, −3) (positive shift) and Z (3, 1) (negative increase) (*p* < 0.0262) (Figure 4D). For the anterior corneal surface, both groups exhibited a significant increase in Z (3, −3) (positively) and Z (4, −4) (negatively), while only the temporal group showed a significant increase in Z (3, 1) and Z (4, 2) (Figure 4E). Additionally, there was a significant decrease in Z (3, 3) (*p* < 0.0262) in the temporal CCI group but an opposite trend with a significant increase (*p* < 0.0167) was observed in the superior CCI group (Figure 4E). For the posterior corneal surface analysis depicted by Figure 4F, both groups demonstrated a significant increase in Z (3, −3) (negatively) and Z (4, −4) (positively), while both groups also showed a notable decrease in Z (4, 4). Furthermore, an increase of Z (3, 3) (*p* < 0.00001) was only observed in the superior CCI group, and a change of Z (4, 2) (*p* = 0.001) only occurred within the temporal CCI group (Figure 4F).

### Correlation analysis between corneal SIA and CCI features

**Figure 5:**
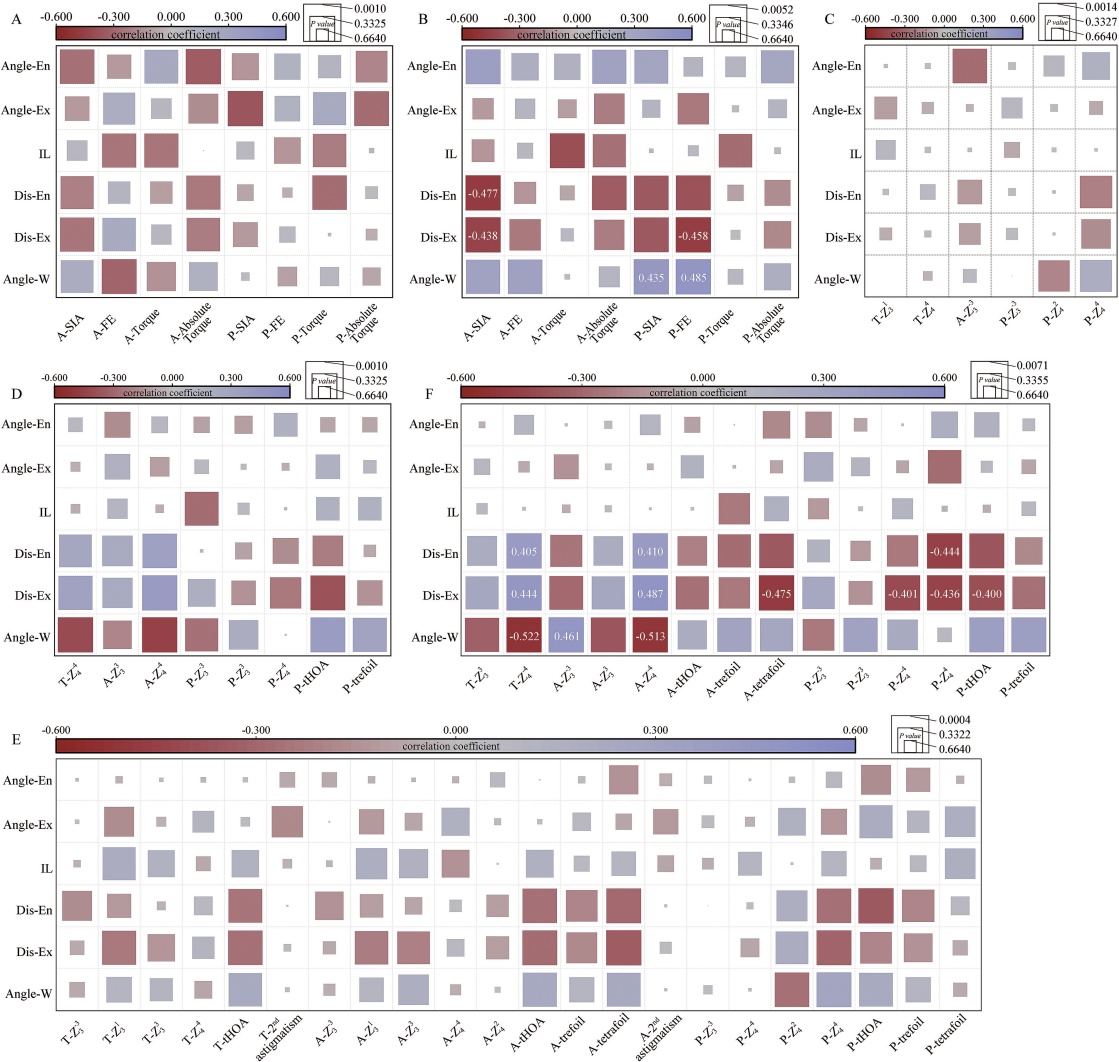
Correlation analysis between surgically induced astigmatism (SIA), corneal higher order aberrations (HOAs), and clear corneal incision (CCI) features. The correlation coefficient is represented by the color of the square, while the *p*-value is indicated by the area of the square. Correlation coefficients with significant *p*-values are annotated in the squares. **(A)** Correlation analysis between SIA and CCI features in the temporal CCI group, the cut off for significant correlation was *p* < 0.0010 after correction for multiple comparisons based on false discovery rate (FDR) (*n* = 48); **(B)** Correlation analysis between SIA and CCI features in the superior CCI group, the cut off for significant correlation was *p* < 0.0052 after correction for multiple comparisons based on FDR (*n* = 48); **(C)** Correlation analysis between corneal HOAs (4-mm zone) and CCI features in the temporal CCI group, the cut off for significant correlation was *p* < 0.0014 after correction for multiple comparisons based on FDR (*n* = 38); **(D)** Correlation analysis between corneal HOAs (4-mm zone) and CCI features in the superior CCI group, the cut off for significant correlation was *p* < 0.0010 after correction for multiple comparisons based on FDR (*n* = 42); **(E)** Correlation analysis between corneal HOAs (6-mm zone) and CCI features in the temporal CCI group, the cut off for significant correlation was *p* < 0.0004 after correction for multiple comparisons based on FDR (*n* = 132); **(F)** Correlation analysis between corneal HOAs (6-mm zone) and CCI features in the superior CCI group, the cut off for significant correlation was *p* < 0.0071 after correction for multiple comparisons based on FDR (*n* = 84). Angle-W, angle corresponding to the corneal incision (incision width); Dis-Ex, the distance from incision exit to central cornea; Dis-En, the distance from incision entry to central cornea; IL, the straight distance from incision entry to exit (incision length); Angle-Ex, angle between incision and corneal endothelium; Angle-En, angle between the incision and corneal epithelium; A, anterior corneal surface; P, posterior corneal surface; T, total cornea.

With an FDR level of 0.05, the cut-off for a significant correlation between SIA and CCI features was 0.0010 in the temporal group and 0.0052 in the superior group. In the temporal CCI group, no significant correlation was observed between SIA and CCI features (Figure 5A). However, in the superior CCI group, there were negative correlations found between anterior corneal SIA magnitude and Dis-Ex (*r* = −0.438, *p* = 0.002), as well as Dis-En (*r* = −0.477, *p* < 0.001) (Figure 5B). Moreover, positive correlations were observed between posterior corneal SIA magnitude (*r* = 0.435, *p* = 0.002) and flattening effect (*r* = 0.485, *p* < 0.001) with Angle-W; whereas a negative correlation was noted between Dis-Ex and posterior corneal flattening effect (*r* = −0.458, *p* = 0.001) (Figure 5B).

### Correlation analysis between corneal HOAs and CCI features

#### 4-mm analytical zone

Only the corneal HOAs that showed statistically significant changes postoperatively were included in the correlation analysis. After correction for multiple comparisons using the FDR, a significant level of *p* < 0.0014 and *p* < 0.0010 was considered statistically significant in the temporal and superior CCI groups, respectively. No statistically significant correlation was found between 4-mm corneal HOAs and CCI features (Figures 5C,D).

#### 6-mm analytical zone

Only the statistically significant changes in corneal HOAs postoperatively were used for correlation analysis. With an FDR level of 0.05, the threshold for significant correlation between 6-mm corneal HOAs and CCI features was determined to be 0.0004 in the temporal CCI group and 0.0071 in the superior CCI group. No significant correlation was observed between corneal HOAs and CCI features in the temporal CCI group (Figure 5E). However, in the superior CCI group, Angle-W exhibited a significant positive correlation with anterior corneal Z (3, −3) (*r* = 0.461, *p* = 0.0002), while showing a significant negative correlation with total (*r* = −0.522, *p* = 0.0011) and anterior (*r* = −0.513, *p* = 0.0002) corneal Z (4, −4) (Figure 5F). The Dis-Ex demonstrated a significant positive correlation with total (*r* = 0.444, *p* = 0.0018) and anterior (*r* = 0.487, *p* = 0.0005) corneal Z (4, –4), but showed a significant negative correlation with anterior corneal tetrafoil (*r* = −0.475, *p* = 0.0007) and posterior corneal Z (4, –4) (*r* = −0.401, *p* = 0.0052), Z (4, 4) (*r* = 0.436, *p* = 0.0022) and tHOAs (*r* = −0.400, *p* = 0.0053) (Figure 5F). Additionally, the Dis-En displayed a significant positive correlation with total (*r* = 0.405, *p* = 0.0048) and anterior (*r* = 0.410, *p* = 0.0042) corneal Z (4, −4) and also had a significant negative correlation with posterior corneal Z (4, 4) (*r* = −0.444, *p* = 0.0018) (Figure 5F).

## Discussion

Temporal and superior CCIs are commonly used CCI locations during ICL implantation in clinical practice. Consistent with the previous study (10), our data validated the satisfactory safety and efficacy of ICL implantation in both temporal and superior CCI groups (Supplementary Figure S1). Given that a majority of patients underwent ICL implantation for high myopia, postoperative CDVA consistently surpassed preoperative CDVA, owing to the elimination of image reduction caused by glasses wear.

Corneal SIA caused by CCI during ICL surgery is inevitable, which may affect postoperative visual and refractive outcomes. SIA resulting from CCI can be influenced by multiple factors, including surgeon’s experience, incision locations, incision width, IL, Dis-Ex, etc. (13, 28–31). It is widely accepted that the magnitude of corneal SIA is positively correlated with CCI width and IL, while negatively correlated with Dis-Ex (11–13). Our data indicated that longer Dis-Ex and Dis-En were observed in the temporal CCI group compared to the superior CCI group due to an oblate shape of the anterior corneal surface (Table 2). Since both groups used keratome with identical widths, shorter Dis-Ex and Dis-En resulted in a larger Angle-W in the superior CCI group (Table 2). The negative correlation between corneal SIA and Dis-Ex has been reported by previous studies (12, 13). The results of our investigation revealed analogous corneal SIA outcomes in both the temporal and superior CCI groups (as shown in Table 2), and a negative correlation between anterior corneal SIA and Dis-Ex/En was observed solely in the superior CCI group. An explanation could be that the temporal corneal CCI was located at a considerable distance from the corneal apex, causing only a minor alteration in corneal SIA. Moreover, our prior research determined that increased CCI length combined with reduced angles of incision entry and exit could lead to higher SIA values on the anterior and posterior corneal surfaces (12). However, no such typical correlation was observed in this study. This can be ascribed to the discrepancy of surgical maneuver and invasion between ICL and cataract surgery, as no phacoemulsification and I/A procedures are required in ICL surgery, leading to the better uniformity of CCI morphology and hence minor correlations to corneal SIA.

In line with the previous report (10), our data also showed a significant increase in corneal astigmatism in the temporal CCI group and decreased astigmatism in the superior CCI group following ICL implantation (Supplementary Table S1). Most ICL candidates are younger patients, who predominantly have with-the-rule astigmatism. Thus, the superior CCI certainly can ameliorate the corneal postoperative with-the-rule astigmatism, compared with the temporal CCI. Nevertheless, it should be noted that corneal astigmatism can be influenced by other factors such as palpebral fissure size and eyelid compression (32). Squinting caused by refractive errors or tension can lead to a substantial increase in vertical corneal refractive power (32). Correcting ocular refractive error, followed by the natural widening of the palpebral fissure, may cause a decrease in the vertical meridian corneal diopter. Caution should be taken when planning the ICL astigmatism and CCI locations for patients with relatively smaller palpebral fissures or corneal diameters in order to achieve optimal visual and refractive outcomes.

Some patients still experience halo symptoms after ICL surgery (33). In addition to the ICL central hole, the increase of HOAs is the significant reason for postoperative halo (34, 35). Previous studies have reported a significant increase in total HOAs, trefoil, and coma aberration as well as a decrease in SA (negative increase) after ICL implantation (34, 36–39). The substantial reduction in SA was mainly due to the negative SA of the ICL itself that increased with its diopter (40), while the changes in coma and trefoil may be associated with the corneal incision (41).

In our patients, both temporal and superior CCI groups showed stable corneal tHOAs, coma, trefoil, tetrafoil, and 2nd astigmatism over the 4-mm optical zone, except for posterior corneal tHOAs and trefoil (Figure 3). However, for the 6-mm optical zone, significant changes of corneal tHOAs, trefoil, and tetrafoil in both groups, along with 2nd astigmatism in the temporal CCI group, were noted (Figure 3). These findings should partially contribute to the night vision symptoms experienced by most patients postoperatively.

For the 6-mm optical zone Z (3, 3) Zernike term, the two groups exhibited opposing changing patterns, while for 4-mm optical zone Z (3, 3), significant changes were only noted in the superior CCI group (Figure 4). No significant correlation between corneal Z (3, 3) and CCI features was found in both groups (Figure 5). These results suggested that the changes of corneal Z (3, 3) seemed to be mainly influenced by CCI locations, not CCI morphological features. The superior CCI potentially introduces more corneal Z (3, 3) and more postoperative corneal aberrations, as Z (3, 3) is an important HOAs term influencing optical quality.

Another important Zernike third-order term [Z (3, 1)] showed a significant increase in the temporal CCI group (Figure 4). It is understandable that the temporal CCI can affect the horizontal corneal coma, evidenced by the remarkable decrease of anterior corneal K_f_ in the temporal CCI group (Supplementary Table S1). On the other hand, the superior CCI group showed no significant coma changes, consistent with the insignificant changes of anterior corneal K_s_ or K_f_ in the superior CCI group (Supplementary Table S1). The underlying mechanism ought to be complex, including the additional effects caused by upper eyelid cover or compression as described above, besides the incision relaxing effect itself. Therefore, compared with the temporal CCI, the superior CCI location might be helpful to minimize the introduction of postoperative coma.

Similarly, only the superior CCI group displayed a connection between corneal HOAs and CCI features. The shorter Dis-Ex/En result was closely related to the changes of Z (4, −4) and Z (4, 4). An increase in the Angle-W resulted in higher values for corneal Z (3, −3) and Z (4, −4) (Figure 5F). The findings suggested a potential link between post-surgery corneal HOAs and specific characteristics of CCI such as Angle-W, Dis-En, and Dis-Ex, particularly when the incision was located superiorly. Incorporating an extended Dis-Ex/En and a diminished Angle-W in the CCI design could be beneficial in decreasing corneal HOAs following ICL implantation.

When determining the optimal placement of CCIs for ICL surgery, both superior and temporal incisions offer unique benefits and challenges. The selection of incision site should be guided by a comprehensive assessment of factors that impact postoperative success and complication risk. Superior incisions are advantageous for correcting pre-existing with-the-rule astigmatism, particularly when non-toric ICLs are used. By strategically placing the incision to flatten the steeper corneal meridian, superior incisions can effectively address astigmatism (42). They may also be less influenced by eyelid movements, which can result in reduced postoperative discomfort, enhanced wound stability, and accelerated healing. Furthermore, the support provided by the patient’s forehead can be beneficial for less experienced surgeons (43). However, superior incisions carry a higher risk of postoperative ocular surface fluid entering the anterior chamber, potentially increasing the likelihood of infection and inflammation (44). Conversely, temporal incisions provide better access in patients with prominent brows or deep-set eyes and are less likely to induce astigmatism, making them an attractive option for patients with minimal pre-existing astigmatism (45). However, temporal incisions may be less effective in astigmatism correction compared to superior incisions and are more susceptible to exposure-related complications. The choice between superior and temporal CCIs should be tailored to the individual patient’s ocular anatomy, the presence of pre-existing astigmatism, and the surgeon’s expertise and preference.

There are several limitations in this study. Firstly, compared with the ICL, more repetitive checking and adjusting maneuvers were performed to ensure accurate alignment of the TICL axis with the intended orientation during TICL implantation, which might have a subtle influence on the corneal contour. Secondly, there were discrepancies in the angles of the central point of CCI (*β*) within the superior and temporal groups (Figure 1B), potentially introducing a bias in the analysis. Therefore, conducting additional research with a larger number of participants could lead to more conclusive findings. Thirdly, the single-center observational retrospective design employed in our study is a significant limitation. To strengthen our conclusions, it is imperative to conduct prospective multi-center studies with larger sample sizes in the future. Lastly, our study was limited in scope as it primarily focused on objective assessments of corneal SIA and HOAs, with visual acuity being the sole subjective measure. Given that patients may encounter a range of visual symptoms beyond mere changes in visual acuity, such as glare, halos, night vision difficulties, and fluctuating vision, our analysis did not encompass the full spectrum of postoperative visual experiences. Further research is required to investigate the correlation between the location of CCIs and ocular HOAs, Also, the effect of CCI location on visual quality, as quantified through questionnaires, warrants further investigation to provide a more holistic understanding of the implications of incision site selection on overall visual performance and patient satisfaction.

Collectively, the corneal SIA after ICL implantation was negatively correlated with Dis-En and Dis-Ex and was comparable in magnitude between the 3.0-mm temporal and superior CCIs. The Zernike 3rd and 4th order terms were significantly associated with the CCI location along with specific CCI characteristics, especially in the superior CCI group. Our findings indicated that the CCI location design can cause subtle differences in postoperative corneal SIA and HOAs. For better corneal optical quality and optimal visual outcomes post-ICL surgery, it is advantageous to have a CCI that is distanced further from the corneal apex, irrespective of whether it is located superiorly or temporally.

## Data Availability

The original contributions presented in the study are included in the article/Supplementary material, further inquiries can be directed to the corresponding author.

## Supplementary material

Supplementary Figure S1 Visual and refractive outcomes after ICL implantation in both temporal and superior clear corneal incision (CCI) groups. **(A)** Attempted versus achieved spherical equivalent refraction (SER) in the temporal CCI group; **(B)** Attempted versus achieved SER in the superior CCI group; **(C)** Cumulative preoperative corrected distance visual acuity (preop-CDVA), postoperative uncorrected distance visual acuity (postop-UDVA), and postoperative corrected distance visual acuity (postop-CDVA) in the temporal CCI group; **(D)** Cumulative preop-CDVA, postop-UDVA, and postop-CDVA in the temporal CCI group; **(E)** The distribution of postoperative SER in the temporal CCI group; **(F)** The distribution of postoperative SER in the temporal CCI group.

Supplementary Figure S2 Corneal surgically induced astigmatism (SIA) on anterior **(A)** and posterior **(B)** corneal surfaces in both temporal (red dots) and superior (blue dots) clear corneal incision (CCI) groups. The red stars denote centroid SIA in the temporal CCI group and the blue stars denote centroid SIA in the superior CCI group.
