# Supplementary figures and images for: Effects of clear corneal incision location and morphology on corneal surgically induced astigmatism and higher-order aberrations after ICL V4c implantation
Source: Front Med (Lausanne). 2024 Nov 6;11:1491901. doi: 10.3389/fmed.2024.1491901 (PMC11576198; doi:10.3389/fmed.2024.1491901)

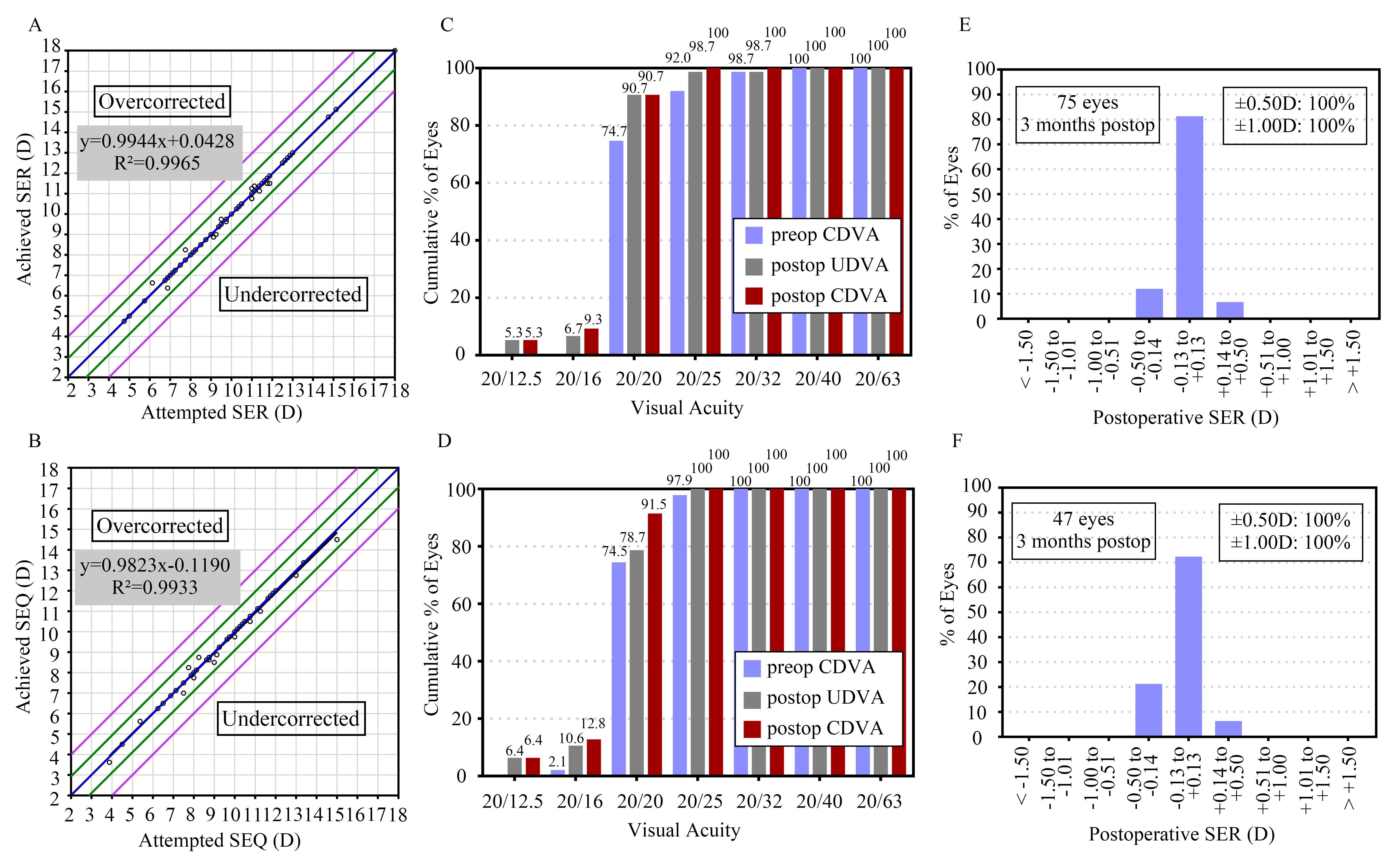

Supplement: Supplementary Figure S1 — Visual and refractive outcomes after ICL implantation in both temporal and superior clear corneal incision (CCI) groups. (A) Attempted versus achieved spherical equivalent refraction (SER) in the temporal CCI group; (B) Attempted versus achieved SER in the superior CCI group; (C) Cumulative preoperative corrected distance visual acuity (preop-CDVA), postoperative uncorrected distance visual acuity (postop-UDVA), and postoperative corrected distance visual acuity (postop-CDVA) in the temporal CCI group; (D) Cumulative preop-CDVA, postop-UDVA, and postop-CDVA in the temporal CCI group; (E) The distribution of postoperative SER in the temporal CCI group; (F) The distribution of postoperative SER in the temporal CCI group. [file Image_1.JPEG]

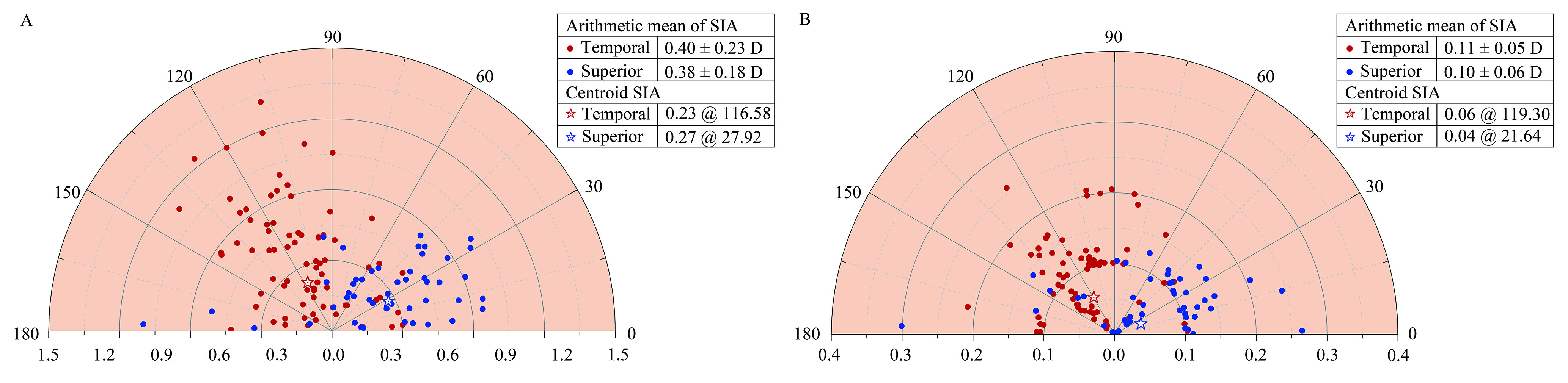

Supplement: Supplementary Figure S2 — Corneal surgically induced astigmatism (SIA) on anterior (A) and posterior (B) corneal surfaces in both temporal (red dots) and superior (blue dots) clear corneal incision (CCI) groups. The red stars denote centroid SIA in the temporal CCI group and the blue stars denote centroid SIA in the superior CCI group. [file Image_2.JPEG]
