# Supplementary material for: Effects of clear corneal incision location and morphology on corneal surgically induced astigmatism and higher-order aberrations after ICL V4c implantation
Source: Front Med (Lausanne). 2024 Nov 6;11:1491901. doi: 10.3389/fmed.2024.1491901 (PMC11576198; doi:10.3389/fmed.2024.1491901)
Supplement: Supplementary file 3 [file Table_1.DOCX]

**Supplemental Table 1** Corneal keratometry data in both groups pre- and postoperatively

|  | | | preoperative | postoperative | *P* value |
| --- | --- | --- | --- | --- | --- |
| Temporal CCI group | | |  |  |  |
|  | Anterior corneal surface | |  |  |  |
|  |  | Kf (D) | 42.79 ± 1.72 | 42.68 ± 1.71 | < 0.00001^*^ |
|  |  | Ks (D) | 44.30 ± 1.72 | 44.34 ± 1.74 | 0.176 |
|  |  | Astigmatism magnitude (D) | 1.51 ± 0.69 | 1.66 ± 0.66 | < 0.00001^*^ |
|  | Posterior corneal surface | |  |  |  |
|  |  | Kf (D) | -6.12 ± 0.28 | -6.08 ± 0.27 | < 0.00001^*^ |
|  |  | Ks (D) | -6.51 ± 0.29 | -6.51 ± 0.29 | 0.373 |
|  |  | Astigmatism magnitude (D) | 0.39 ± 0.15 | 0.43 ± 0.14 | < 0.0001^*^ |
| Superior CCI group | | |  |  |  |
|  | Anterior corneal surface | |  |  |  |
|  |  | Kf (D) | 43.12 ± 1.81^b^ | 43.10 ± 1.77^b^ | 0.602 |
|  |  | Ks (D) | 44.34 ± 1.69^b^ | 44.29 ± 1.71^b^ | 0.149 |
|  |  | Astigmatism magnitude (D) | 1.22 ± 0.69^b^ | 1.18 ± 0.61^a^ | 0.294 |
|  | Posterior corneal surface | |  |  |  |
|  |  | Kf (D) | -6.14 ± 0.27^b^ | -6.13 ± 0.27^b^ | 0.200 |
|  |  | Ks (D) | -6.49 ± 0.29^b^ | -6.44 ± 0.28^b^ | < 0.0001^†^ |
|  |  | Astigmatism magnitude (D) | 0.35 ± 0.12^b^ | 0.31 ± 0.13^a^ | 0.001^†^ |

CCI = clear corneal incision, D = diopter, K_f_: flat keratometry, K_s_ = steep keratometry

^*^significant (*P* < 0.0417) after correction for multiple comparisons (n = 6) based on false discovery rate (FDR) in the temporal CCI group;

^†^significant (*P* < 0.0250) after correction for multiple comparisons (n = 6) based on FDR in the superior CCI group.

^a^significant after correction for multiple comparisons (n = 8) based on FDR for preoperative (*P* < 0.0083) and postoperative (*P* < 0.125) group-wise comparison.

^b^no significant defference after correction for multiple comparisons (n = 6) based on FDR for preoperative (*P* > 0.0083) and postoperative (*P* > 0.0125) group-wise comparison.
