# Supplementary material for: Effects of clear corneal incision location and morphology on corneal surgically induced astigmatism and higher-order aberrations after ICL V4c implantation
Source: Front Med (Lausanne). 2024 Nov 6;11:1491901. doi: 10.3389/fmed.2024.1491901 (PMC11576198; doi:10.3389/fmed.2024.1491901)
Supplement: Supplementary file 4 [file Table_2.DOCX]

**Supplemental Table 2 Corneal HOAs over 4-mm zone of both temporal and superior CCI groups preoperatively**

|  | | temporal | superior | *P* value |
| --- | --- | --- | --- | --- |
| Total cornea | | | | |
|  | Z(3,-3) | 0.00 ± 0.04 | 0.00 ± 0.04 | 0.939 |
|  | Z(3,-1) | -0.02 ± 0.07 | -0.03 ± 0.06 | 0.355 |
|  | Z(3,1) | -0.01 ± 0.04 | -0.02 ± 0.05 | 0.105 |
|  | Z(3,3) | 0.00 ± 0.03 | 0.00 ± 0.03 | 0.962 |
|  | Z(4,-4) | -0.01 ± 0.03 | 0.00 ± 0.02 | 0.116 |
|  | Z(4,-2) | 0.00 ± 0.02 | -0.01 ± 0.02 | 0.977 |
|  | Z(4,0) | 0.02 ± 0.03 | 0.01 ± 0.03 | 0.224 |
|  | Z(4,2) | -0.01 ± 0.02 | -0.01 ± 0.02 | 0.234 |
|  | Z(4,4) | -0.02 ± 0.03 | -0.02 ± 0.04 | 0.359 |
|  | tHOAs | 0.11 ± 0.04 | 0.11 ± 0.05 | 0.795 |
|  | Trefoil | 0.04 ± 0.02 | 0.04 ± 0.03 | 0.509 |
|  | Coma | 0.07 ± 0.04 | 0.07 ± 0.05 | 0.889 |
|  | Tetrafoil | 0.03 ± 0.02 | 0.04 ± 0.03 | 0.780 |
|  | 2^nd^ astigmatism | 0.03 ± 0.02 | 0.03 ± 0.02 | 0.595 |
| Anterior corneal surface | | | | |
|  | Z(3,-3) | 0.00 ± 0.03 | 0.00 ± 0.03 | 0.674 |
|  | Z(3,-1) | -0.02 ± 0.07 | -0.03 ± 0.06 | 0.419 |
|  | Z(3,1) | -0.02 ± 0.03 | -0.03 ± 0.04 | 0.089 |
|  | Z(3,3) | 0.00 ± 0.03 | -0.01 ± 0.03 | 0.566 |
|  | Z(4,-4) | 0.00 ± 0.02 | 0.00 ± 0.02 | 0.075 |
|  | Z(4,-2) | 0.00 ± 0.01 | 0.00 ± 0.01 | 0.127 |
|  | Z(4,0) | 0.03 ± 0.03 | 0.03 ± 0.03 | 0.438 |
|  | Z(4,2) | -0.01 ± 0.02 | 0.00 ± 0.02 | 0.571 |
|  | Z(4,4) | 0.00 ± 0.02 | 0.00 ± 0.03 | 0.879 |
|  | tHOAs | 0.11 ± 0.03 | 0.11 ± 0.04 | 0.638 |
|  | Trefoil | 0.04 ± 0.02 | 0.04 ± 0.02 | 0.536 |
|  | Coma | 0.07 ± 0.04 | 0.07 ± 0.04 | 0.914 |
|  | Tetrafoil | 0.03 ± 0.02 | 0.03 ± 0.02 | 0.933 |
|  | 2^nd^ astigmatism | 0.02 ± 0.01 | 0.02 ± 0.01 | 0.941 |
| Posterior corneal surface | | | | |
|  | Z(3,-3) | 0.00 ± 0.02 | 0.00 ± 0.02 | 0.391 |
|  | Z(3,-1) | 0.00 ± 0.02 | 0.00 ± 0.02 | 0.651 |
|  | Z(3,1) | 0.00 ± 0.01 | 0.01 ± 0.01 | 0.352 |
|  | Z(3,3) | 0.01 ± 0.02 | 0.01 ± 0.01 | 0.647 |
|  | Z(4,-4) | 0.00 ± 0.01 | 0.00 ± 0.01 | 0.856 |
|  | Z(4,-2) | 0.00 ± 0.01 | 0.00 ± 0.01 | 0.094 |
|  | Z(4,0) | -0.03 ± 0.02 | -0.03 ± 0.02 | 0.801 |
|  | Z(4,2) | 0.00 ± 0.01 | 0.00 ± 0.01 | 0.073 |
|  | Z(4,4) | -0.02 ± 0.01 | -0.02 ± 0.01 | 0.022 |
|  | tHOAs | 0.06 ± 0.01 | 0.06 ± 0.01 | 0.328 |
|  | Trefoil | 0.02 ± 0.01 | 0.02 ± 0.01 | 0.535 |
|  | Coma | 0.02 ± 0.01 | 0.02 ± 0.01 | 0.502 |
|  | Tetrafoil | 0.02 ± 0.01 | 0.02 ± 0.01 | 0.227 |
|  | 2^nd^ astigmatism | 0.01 ± 0.01 | 0.01 ± 0.01 | 0.925 |

CCI = clear corneal incision, tHOAs = total higher-order aberrations

With an FDR level of 0.05 (n = 42), the cut-off for significant difference between temporal and superior group was *P* < 0.0012.
