# Supplementary material for: Effects of clear corneal incision location and morphology on corneal surgically induced astigmatism and higher-order aberrations after ICL V4c implantation
Source: Front Med (Lausanne). 2024 Nov 6;11:1491901. doi: 10.3389/fmed.2024.1491901 (PMC11576198; doi:10.3389/fmed.2024.1491901)
Supplement: Supplementary file 5 [file Table_3.DOCX]

**Supplemental Table 3 Corneal HOAs over 6-mm zone of both temporal and superior CCI groups preoperatively**

|  | | temporal | superior | *P* value |
| --- | --- | --- | --- | --- |
| Total cornea | | | | |
|  | Z(3,-3) | -0.01 ± 0.11 | -0.03 ± 0.11 | 0.301 |
|  | Z(3,-1) | -0.05 ± 0.20 | -0.04 ± 0.19 | 0.825 |
|  | Z(3,1) | -0.06 ± 0.09 | -0.10 ± 0.11 | 0.043 |
|  | Z(3,3) | -0.02 ± 0.08 | -0.02 ± 0.08 | 0.951 |
|  | Z(4,-4) | 0.01 ± 0.07 | 0.02 ± 0.07 | 0.387 |
|  | Z(4,-2) | -0.02 ± 0.04 | -0.02 ± 0.04 | 0.554 |
|  | Z(4,0) | 0.18 ± 0.08 | 0.17 ± 0.10 | 0.723 |
|  | Z(4,2) | -0.03 ± 0.06 | -0.02 ± 0.07 | 0.398 |
|  | Z(4,4) | -0.06 ± 0.07 | -0.07 ± 0.09 | 0.570 |
|  | tHOAs | 0.36 ± 0.11 | 0.38 ± 0.11 | 0.375 |
|  | Trefoil | 0.12 ± 0.06 | 0.13 ± 0.05 | 0.176 |
|  | Coma | 0.20 ± 0.12 | 0.22 ± 0.12 | 0.294 |
|  | Tetrafoil | 0.10 ± 0.05 | 0.11 ± 0.08 | 0.768 |
|  | 2^nd^ astigmatism | 0.07 ± 0.04 | 0.07 ± 0.05 | 0.937 |
| Anterior corneal surface | | | | |
|  | Z(3,-3) | 0.01 ± 0.10 | -0.02 ± 0.10 | 0.149 |
|  | Z(3,-1) | -0.03 ± 0.20 | -0.03 ± 0.18 | 0.987 |
|  | Z(3,1) | -0.06 ± 0.09 | -0.09 ± 0.11 | 0.056 |
|  | Z(3,3) | -0.03 ± 0.08 | -0.03 ± 0.08 | 0.924 |
|  | Z(4,-4) | 0.01 ± 0.05 | 0.02 ± 0.06 | 0.338 |
|  | Z(4,-2) | -0.02 ± 0.04 | -0.02 ± 0.03 | 0.112 |
|  | Z(4,0) | 0.23 ± 0.07 | 0.23 ± 0.10 | 0.795 |
|  | Z(4,2) | -0.02 ± 0.05 | -0.01 ± 0.06 | 0.925 |
|  | Z(4,4) | -0.02 ± 0.06 | -0.02 ± 0.08 | 0.889 |
|  | tHOAs | 0.37 ± 0.10 | 0.38 ± 0.11 | 0.750 |
|  | Trefoil | 0.12 ± 0.07 | 0.12 ± 0.05 | 0.311 |
|  | Coma | 0.19 ± 0.12 | 0.20 ± 0.11 | 0.497 |
|  | Tetrafoil | 0.07 ± 0.04 | 0.08 ± 0.06 | 0.619 |
|  | 2^nd^ astigmatism | 0.06 ± 0.04 | 0.06 ± 0.04 | 0.985 |
| Posterior corneal surface | | | | |
|  | Z(3,-3) | -0.02 ± 0.04 | -0.01 ± 0.05 | 0.228 |
|  | Z(3,-1) | -0.01 ± 0.04 | -0.02 ± 0.04 | 0.513 |
|  | Z(3,1) | 0.00 ± 0.02 | 0.00 ± 0.02 | 0.952 |
|  | Z(3,3) | 0.02 ± 0.03 | 0.02 ± 0.03 | 0.657 |
|  | Z(4,-4) | -0.01 ± 0.03 | -0.01 ± 0.02 | 0.601 |
|  | Z(4,-2) | 0.00 ± 0.01 | 0.00 ± 0.01 | 0.606 |
|  | Z(4,0) | -0.16 ± 0.02 | -0.16 ± 0.03 | 0.777 |
|  | Z(4,2) | -0.01 ± 0.02 | 0.00 ± 0.02 | 0.162 |
|  | Z(4,4) | -0.04 ± 0.03 | -0.05 ± 0.02 | 0.014 |
|  | tHOAs | 0.19 ± 0.03 | 0.19 ± 0.03 | 0.784 |
|  | Trefoil | 0.05 ± 0.03 | 0.05 ± 0.03 | 0.355 |
|  | Coma | 0.04 ± 0.02 | 0.04 ± 0.02 | 0.933 |
|  | Tetrafoil | 0.05 ± 0.03 | 0.06 ± 0.02 | 0.188 |
|  | 2^nd^ astigmatism | 0.02 ± 0.01 | 0.02 ± 0.01 | 0.566 |

CCI = clear corneal incision, tHOAs = total higher order aberrations

With an FDR level of 0.05 (n = 42), the cut-off for significant difference between temporal and superior group was *P* < 0.0012.
