# Supplementary material for: Effects of clear corneal incision location and morphology on corneal surgically induced astigmatism and higher-order aberrations after ICL V4c implantation
Source: Front Med (Lausanne). 2024 Nov 6;11:1491901. doi: 10.3389/fmed.2024.1491901 (PMC11576198; doi:10.3389/fmed.2024.1491901)
Supplement: Supplementary file 6 [file Table_4.DOCX]

**Supplemental Table 4 Corneal HOAs over 4-mm zone of both temporal and superior CCI groups postoperatively**

|  | | temporal | superior | *P* value |
| --- | --- | --- | --- | --- |
| Total cornea | | | | |
|  | Z(3,-3) | 0.00 ± 0.04 | 0.00 ± 0.04 | 0.385 |
|  | Z(3,-1) | -0.03 ± 0.08 | -0.03 ± 0.08 | 0.864 |
|  | Z(3,1) | -0.02 ± 0.04 | -0.02 ± 0.04 | 0.925 |
|  | Z(3,3) | 0.01 ± 0.03 | 0.00 ± 0.03 | 0.867 |
|  | Z(4,-4) | -0.01 ± 0.03 | -0.01 ± 0.02 | 0.960 |
|  | Z(4,-2) | -0.01 ± 0.01 | 0.00 ± 0.02 | 0.514 |
|  | Z(4,0) | 0.02 ± 0.03 | 0.02 ± 0.02 | 0.775 |
|  | Z(4,2) | -0.01 ± 0.02 | -0.01 ± 0.02 | 0.710 |
|  | Z(4,4) | 0.00 ± 0.03 | -0.02 ± 0.03 | 0.004* |
|  | tHOAs | 0.11 ± 0.05 | 0.12 ± 0.04 | 0.452 |
|  | Trefoil | 0.04 ± 0.02 | 0.04 ± 0.02 | 0.724 |
|  | Coma | 0.08 ± 0.05 | 0.08 ± 0.05 | 0.877 |
|  | Tetrafoil | 0.03 ± 0.02 | 0.04 ± 0.02 | 0.175 |
|  | 2^nd^ astigmatism | 0.03 ± 0.02 | 0.03 ± 0.01 | 0.881 |
| Anterior corneal surface | | | | |
|  | Z(3,-3) | 0.01 ± 0.03 | 0.01 ± 0.03 | 0.313 |
|  | Z(3,-1) | -0.03 ± 0.07 | -0.02 ± 0.07 | 0.800 |
|  | Z(3,1) | -0.02 ± 0.03 | -0.03 ± 0.04 | 0.738 |
|  | Z(3,3) | 0.00 ± 0.03 | -0.02 ± 0.03 | 0.001* |
|  | Z(4,-4) | -0.01 ± 0.02 | -0.01 ± 0.02 | 0.482 |
|  | Z(4,-2) | 0.00 ± 0.01 | 0.00 ± 0.02 | 0.528 |
|  | Z(4,0) | 0.04 ± 0.02 | 0.04 ± 0.02 | 0.994 |
|  | Z(4,2) | -0.01 ± 0.02 | -0.01 ± 0.02 | 0.968 |
|  | Z(4,4) | 0.01 ± 0.02 | -0.01 ± 0.03 | 0.030 |
|  | tHOAs | 0.11 ± 0.04 | 0.12 ± 0.04 | 0.329 |
|  | Trefoil | 0.04 ± 0.02 | 0.04 ± 0.02 | 0.819 |
|  | Coma | 0.07 ± 0.05 | 0.07 ± 0.05 | 0.916 |
|  | Tetrafoil | 0.03 ± 0.02 | 0.03 ± 0.02 | 0.937 |
|  | 2^nd^ astigmatism | 0.02 ± 0.01 | 0.02 ± 0.01 | 0.776 |
| Posterior corneal surface | | | | |
|  | Z(3,-3) | -0.02 ± 0.02 | -0.01 ± 0.02 | 0.699 |
|  | Z(3,-1) | 0.00 ± 0.02 | -0.01 ± 0.02 | 0.106 |
|  | Z(3,1) | 0.00 ± 0.01 | 0.01 ± 0.01 | 0.171 |
|  | Z(3,3) | 0.01 ± 0.02 | 0.03 ± 0.02 | 0.000* |
|  | Z(4,-4) | 0.00 ± 0.01 | 0.00 ± 0.01 | 0.017 |
|  | Z(4,-2) | 0.00 ± 0.01 | 0.00 ± 0.00 | 0.878 |
|  | Z(4,0) | -0.04 ± 0.01 | -0.04 ± 0.01 | 0.400 |
|  | Z(4,2) | 0.00 ± 0.01 | 0.00 ± 0.01 | 0.407 |
|  | Z(4,4) | -0.01 ± 0.01 | -0.01 ± 0.02 | 0.034 |
|  | tHOAs | 0.06 ± 0.01 | 0.07 ± 0.01 | 0.001* |
|  | Trefoil | 0.03 ± 0.01 | 0.04 ± 0.01 | 0.000* |
|  | Coma | 0.02 ± 0.01 | 0.02 ± 0.01 | 0.387 |
|  | Tetrafoil | 0.02 ± 0.01 | 0.02 ± 0.01 | 0.006* |
|  | 2^nd^ astigmatism | 0.01 ± 0.00 | 0.01 ± 0.01 | 0.578 |

CCI = clear corneal incision, tHOAs = total higher order aberrations

*: With an FDR level of 0.05 (n = 42), the cut-off for significant difference between temporal and superior group was *P* < 0.0071.
