# Supplementary material for: Effects of clear corneal incision location and morphology on corneal surgically induced astigmatism and higher-order aberrations after ICL V4c implantation
Source: Front Med (Lausanne). 2024 Nov 6;11:1491901. doi: 10.3389/fmed.2024.1491901 (PMC11576198; doi:10.3389/fmed.2024.1491901)
Supplement: Supplementary file 7 [file Table_5.DOCX]

**Supplemental Table 5 Corneal HOAs over 6-mm zone of both temporal and superior CCI groups postoperatively**

|  | | temporal | superior | *P* value |
| --- | --- | --- | --- | --- |
| Total cornea | | | | |
|  | Z(3,-3) | 0.01 ± 0.11 | 0.00 ± 0.13 | 0.571 |
|  | Z(3,-1) | -0.06 ± 0.20 | -0.04 ± 0.20 | 0.508 |
|  | Z(3,1) | -0.09 ± 0.09 | -0.09 ± 0.10 | 0.802 |
|  | Z(3,3) | 0.01 ± 0.10 | -0.06 ± 0.09 | 0.000* |
|  | Z(4,-4) | -0.04 ± 0.08 | -0.05 ± 0.07 | 0.538 |
|  | Z(4,-2) | -0.03 ± 0.04 | -0.02 ± 0.04 | 0.254 |
|  | Z(4,0) | 0.19 ± 0.08 | 0.18 ± 0.09 | 0.879 |
|  | Z(4,2) | -0.03 ± 0.07 | -0.01 ± 0.06 | 0.076 |
|  | Z(4,4) | -0.04 ± 0.08 | -0.07 ± 0.09 | 0.046 |
|  | tHOAs | 0.38 ± 0.12 | 0.40 ± 0.10 | 0.382 |
|  | Trefoil | 0.13 ± 0.06 | 0.15 ± 0.08 | 0.134 |
|  | Coma | 0.21 ± 0.12 | 0.20 ± 0.12 | 0.623 |
|  | Tetrafoil | 0.11 ± 0.07 | 0.13 ± 0.07 | 0.107 |
|  | 2^nd^ astigmatism | 0.08 ± 0.05 | 0.07 ± 0.04 | 0.254 |
| Anterior corneal surface | | | | |
|  | Z(3,-3) | 0.06 ± 0.11 | 0.05 ± 0.14 | 0.486 |
|  | Z(3,-1) | -0.05 ± 0.20 | -0.03 ± 0.19 | 0.547 |
|  | Z(3,1) | -0.09 ± 0.09 | -0.09 ± 0.10 | 0.945 |
|  | Z(3,3) | 0.00 ± 0.11 | -0.11 ± 0.10 | 0.000* |
|  | Z(4,-4) | -0.05 ± 0.07 | -0.06 ± 0.07 | 0.182 |
|  | Z(4,-2) | -0.03 ± 0.03 | -0.02 ± 0.04 | 0.194 |
|  | Z(4,0) | 0.24 ± 0.07 | 0.24 ± 0.09 | 0.977 |
|  | Z(4,2) | -0.03 ± 0.06 | -0.01 ± 0.06 | 0.105 |
|  | Z(4,4) | -0.01 ± 0.07 | -0.04 ± 0.09 | 0.300 |
|  | tHOAs | 0.41 ± 0.11 | 0.44 ± 0.10 | 0.113 |
|  | Trefoil | 0.15 ± 0.07 | 0.18 ± 0.09 | 0.025 |
|  | Coma | 0.20 ± 0.12 | 0.20 ± 0.12 | 0.746 |
|  | Tetrafoil | 0.10 ± 0.06 | 0.12 ± 0.07 | 0.178 |
|  | 2^nd^ astigmatism | 0.07 ± 0.04 | 0.07 ± 0.04 | 0.723 |
| Posterior corneal surface | | | | |
|  | Z(3,-3) | -0.06 ± 0.04 | -0.06 ± 0.05 | 0.438 |
|  | Z(3,-1) | -0.01 ± 0.04 | -0.01 ± 0.04 | 0.736 |
|  | Z(3,1) | 0.01 ± 0.02 | 0.01 ± 0.02 | 0.889 |
|  | Z(3,3) | 0.01 ± 0.05 | 0.07 ± 0.04 | 0.000* |
|  | Z(4,-4) | 0.01 ± 0.02 | 0.02 ± 0.02 | 0.008 |
|  | Z(4,-2) | 0.00 ± 0.01 | 0.00 ± 0.01 | 0.291 |
|  | Z(4,0) | -0.16 ± 0.02 | -0.16 ± 0.03 | 0.840 |
|  | Z(4,2) | 0.00 ± 0.02 | 0.00 ± 0.02 | 0.482 |
|  | Z(4,4) | -0.03 ± 0.03 | -0.04 ± 0.03 | 0.077 |
|  | tHOAs | 0.20 ± 0.03 | 0.22 ± 0.04 | 0.023 |
|  | Trefoil | 0.08 ± 0.04 | 0.10 ± 0.04 | 0.023 |
|  | Coma | 0.04 ± 0.02 | 0.04 ± 0.03 | 0.168 |
|  | Tetrafoil | 0.04 ± 0.02 | 0.05 ± 0.02 | 0.004* |
|  | 2^nd^ astigmatism | 0.02 ± 0.01 | 0.02 ± 0.01 | 0.956 |

CCI = clear corneal incision, tHOAs = total higher order aberrationgs

*: With an FDR level of 0.05 (n = 42), the cut-off for significant difference between temporal and superior group was *P* < 0.0048.
